# Caregivers' active role in palliative home care – to encourage or to dissuade? A qualitative descriptive study

**DOI:** 10.1186/1472-684X-7-15

**Published:** 2008-09-16

**Authors:** Anna Weibull, Frede Olesen, Mette Asbjoern Neergaard

**Affiliations:** 1 General Practice, Grenaa, Denmark; 2 Research Unit for General Practice, Department of Public Health, University of Aarhus, Denmark

## Abstract

**Background:**

Spouses' involvement in palliative care is often a prerequisite for home death, but it is unclear whether active involvement of the spouse, e.g. administering and being in charge of oral or subcutaneous medication or taking care of the patient's personal hygiene, could be harmful or have negative effects on the spouse's experience of the palliative course of disease. The aim of this study was to explore the impact of bereaved spouses' active involvement in medical and physical care on their experience of the palliative course of disease.

**Methods:**

The study was a qualitative, descriptive study based on semi-structured individual interviews with seven bereaved spouses.

**Results:**

Four main categories were found: Degree of involvement, Positive and Negative impact and Prerequisites. The prerequisites found for a positive outcome were Safety (24-hour back-up), Confidence (Professionals' confidence in the spouses' abilities) and Dialog (Spouses' influence on decision-making and being asked).

**Conclusion:**

The results from this study identified important issues whenever spouses take an active part in medical treatment and physical care of critically ill patients in palliative care. The results question the previous research that active involvement of family care givers could be harmful and add preconditions to a positive outcome. More research into these preconditions is needed.

## Background

Family members have always been active in palliative home care, but in modern times death is no longer merely a family event. The role of the professional extends into the home of the dying, where primary health care and palliative care teams can provide highly specialized treatment for the dying. The spouse's involvement in this treatment is often a prerequisite for home death.

Home deaths have been shown to be associated with both better bereavement response and better physical health post-bereavement than inpatient deaths [1] and it has been shown to be important to the family member to have been doing something for the loved one who died [2-4].

Family caregivers seem to cope better with bereavement after a loved one's death if sufficient support is provided during the palliative stage of disease at home [5-12].

However, literature has shown that whenever family caregivers take an active part in the patients' treatment it might have a negative effect on the relatives' mental and physical wellbeing [2,13] and that the relatives sometimes experience an unspoken pressure from the professionals to be 'professionals' rather than loving relatives [5].

Hence, we do not know in which situations active involvement of the spouse, e.g. administering and being in charge of oral or subcutaneous medication or taking care of the patient's personal hygiene (table 1), could be harmful or have a negative impact on their experience of the palliative course of disease. How do we as professionals in palliative home care strike a balance between the spouses' wish to help their loved ones with all the positive effects it might have on the patient and spouse and the spouses' resources with the negative effects it has to exceed their personal limits?

**Table 1:** Active involvement

Examples of family caregivers' active involvement in palliative care	Being in charge of the patients medication
	Administering oral or subcutaneous medication
	Administering subcutaneous needles
	Personal hygiene of the patient
	Treatment of wounds
	Dealing with ostomy, stomach tube etc.

Explanation of the term "active involvement" used in this study

It follows from this that it is unclear whether the health care professionals should actually encourage and recommend the spouses to take an active, hands-on role, in palliative care. Furthermore we need to know which conditions concerning the active involvement of the spouses should be fulfilled to secure a positive outcome for the spouses and the patients.

Hence, there is a profound need for greater understanding of how and if the professionals should approach the family caregivers concerning active involvement in medical and physical care.

The aim of this study was to explore the impact that bereaved spouses' active involvement in medical and physical care has on their experience of the palliative course of disease.

## Methods

This study was a qualitative, descriptive study based on semi-structured individual interviews with seven bereaved spouses from an area (with approximately 30,000 inhabitants) of Djursland, Denmark from December 2006 to March 2007.

### Setting

The Danish health care system is financed through taxes and provides free and equal access to health care services. More than 98% of the Danes are registered with a general practitioner (GP) and receive free medical care [14]. Danish GPs provide most of the health care themselves and act as gatekeepers for access to specialist treatment.

Palliative home care in Denmark is performed by the local GP to whom the patient is registered, the primary health care nurses and the palliative care teams organized at the hospitals. In the study area, the distance to the local hospital is up to 60 km and there is hardly any access to home visits by the palliative care team. Specialist advice can be achieved by the GP or the nurses by telephone contact with the palliative team or by admitting the patient to the hospital. Primary health care nurses visit patients at home on a 24-hour basis. GPs mostly visit patients at home during working hours and some allow their patients to contact them during out-of-clinic hours by providing the caregiver with their private telephone number. Relatives who act as caregivers for terminally ill patients can be granted paid leave from regular jobs by the social security system.

### Sample

The respondents were all spouses of terminally ill cancer patients in a palliative setting at home (Table 2). All 27 GPs in the local area were contacted by E-mail, and informed about the project and its aim. They were asked to refer bereaved spouses fulfilling the inclusion criteria to the authors. Five respondents were referred from GPs and two respondents were identified among the relatives of deceased patients of one of the authors (AW) (Respondent nr 4 and 7, table 2). All spouses asked agreed to participate. The demographics are seen in table 2. The patient male/female rate was 4/3, the average patients' age at time of death was 62 (Range: 53;72) and lung cancer was the dominating cancer type. The spouses' average age at inclusion was 61 (Range: 55;70), three of them had terminal leave, three were on pension and one was unemployed.

**Table 2:** Demographics

**Respondent nr.**	**Deceased patient**			**Family caregiver (Respondent)**				
	
	**Sex**	**Age**	**Diagnosis**	**Relation to patient**	**Sex**	**Age**	**Terminal leave**	**Occupation status**
1	Male	72	Lung cancer	Spouse	Female	63	No	On pension
2	Male	72	Lung cancer	Spouse	Female	70	No	On pension
3	Male	63	Primary tumour unknown	Spouse	Female	55	Yes	Active work
4	Female	56	Pancoast tumour (lung cancer)	Spouse	Male	59	No	Unemployed
5	Female	63	Lung cancer	Spouse	Male	69	No	On pension
6	Male	57	Lung cancer	Spouse	Female	56	Yes	Active work
7	Female	53	Breast cancer	Spouse	Male	56	Yes	Active work

Patient and Respondent demographics

### Ethics

The study was presented to the regional ethics committee of Aarhus County in December 2005. Their answer was that according to Danish committee law (§ 8, stk. 3) all research projects in Denmark involving human beings or any kind of human tissue, cells etc. need permission from a regional ethics committee, but since this study was an interview study and did not involve biological material it did not need permission (Journal-nr: 2005-2.0/85).

The potential informants were informed of the project by letter and were asked to fill a form with demographic information, sign it and send it to the authors if they were interested in participating in the interview. Subsequently, if they met the inclusion criteria they were contacted by phone by AW and an interview was arranged in the home of the spouse. The inclusion criteria were amongst others that the palliative course of disease mainly took place at home and that the time of death of the patient occurred one to three years before the interview (Table 3), implying that the immediate grief was over and a period of recollection had elapsed in order to describe how the events during the palliative course of disease had influenced bereavement. Furthermore by using the GPs who knew the spouses to refer potential respondents to the study, we tried to assure that the respondents were ready in terms of grief to participate in our interview.

**Table 3:** Inclusion criteria

Inclusion criteria for bereaved relatives	The relative must be spouse to an adult deceased cancer patient
	The palliative course of disease must mainly have taken place at home
	The cancer patient must have died between one and three years before the inclusion
	The patient and spouse must be 18 years or more
	The spouse must speak and understand Danish
	The spouse must be mentally and physically able to participate in an interview

### Interviews

All interviews were performed by AW in the homes of the relatives. All authors are GPs; AW is also a qualified specialist in palliative care (Nordic Course in Palliative Medicine 2005–2007). FO and MAN are experienced in qualitative research methods.

A topic guide with open-ended questions designed on the basis of clinical experience, consensus discussions among the authors, and extensive literature studies was used [15]. Main themes were: 1. Description of the course of disease, 2. Feelings connected to active involvement in care giving, 3. Death and bereavement (Table 4). The topic guide was further developed according to the themes emerging during the analysis conducted between the researchers after each interview. The interviews lasted from 65 to 95 minutes.

**Table 4:** Themes

**Main Themes**	**Theme questions**
Description of the course of disease	Who participated?
	What happened?
	How were you involved?
	What was your influence on decisions?
	Who asked you to participate?
	Who helped you?
Feelings connected to active involvement in care giving	What were your positive/negative feelings?
	What were your personal limits?
Death and bereavement	What was the implication on bereavement?
	Would you do it again?
	What were good/bad experiences?
	What is your advice to others?

Themes in topic guide

### Analysis

**Figure 1:**
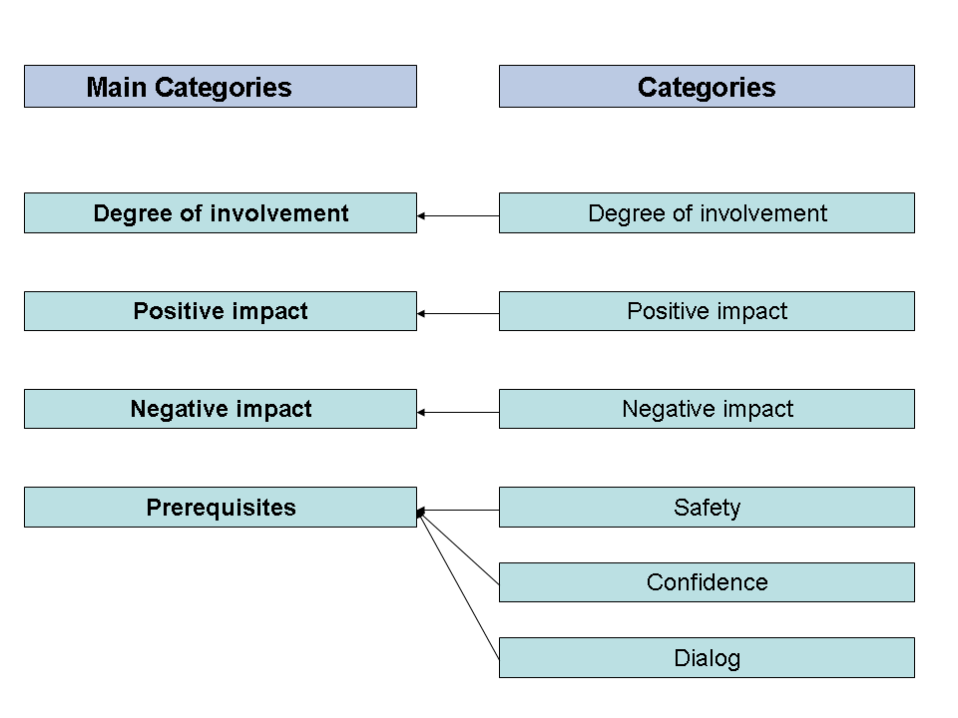
Categories and Main categories.

Digital tape recording followed by verbatim transcription by a trained professional secretary was performed. All researchers read the transcripts thoroughly to get an overall impression of the material before the initial coding [16]. The research approach chosen was Qualitative Description, since we pursued presentation of the facts from the informants' point of view and wanted to stay close to the data [17,18]. Data collection and analysis occurred concurrently. All meaningful text units were identified and coded. The information in each code was compared with other codes and the codes were subsequently grouped into six groups of codes and labelled with a name of a category. The codes from the two interviews with spouses of AW's former patients did not differ in content from the others and were included in the analysis. Again the information in each category was examined and the categories were grouped into four main categories (figure 1) [19]. In this way we allowed the main categories to evolve from the data instead of imposing a framework *a priori *[19]. No new categories emerged from the analysis of the last group interview. The authors agreed on the analysis of the initial coding, the categories, and the main categories. To enhance rigour in our Qualitative Descriptive study we focused on authenticity (The attention to the voices of participants or the ability to remain true to the phenomena under study), credibility (A reflexion of how believable results are), criticality (The critical appraisal of every decision made throughout the research process) and integrity (Demonstrated by on-going reflection and self-criticality of the researcher) [20].

Data coding was performed using the software package NVivo, ed. 6 and triangulation within the group of researchers was used to ensure the quality of coding.

## Results

Four main categories were identified: Degree of involvement, Positive and Negative impact and Prerequisites.

### Degree of involvement

The interviews revealed that all the caregivers had been active in medical and physical treatment of the deceased and they experienced satisfaction having done it. All had been administering medication as required for breakthrough pain and other symptoms either alone or in cooperation with the patient. Three had been extensively active in invasive medical treatment administering subcutaneous needles and medicine. One of these expressed the strain and burden of care giving, but at the same time the positive impact on bereavement.

*"But you see, it is because you have done your very best over a long period of time, isn't it. And it ... well, it is an odd thing to say, but we were bloody tired in the end, I can tell you so ... and I can't...I can't see...she died at home without any problems, and we laid her in the coffin, and we carried her out of the house, didn't we, and we lowered her into the grave – well I can't see... no matter how you look at it or no matter what you do, you can't... it couldn't be... that's the way it was! And after all it feels good to have done it." *(Respondent 7)

The above quote demonstrates that all the respondents went through a period of life where they reached their limits psychologically and physically, and still ended up having had a positive experience due to the fact that they contributed and were able to do so.

All had 24-hour back-up from the primary health care nurses and assistants. All had either terminal leave, were on pension or out of work (Table 1). All had regular contact with the GP, five of them as planned weekly visits at home, and all had visits by the nurse or GP when needed. Six had the private telephone number of their GP to be used when needed in emergencies or if advice was needed regarding medical treatment.

*"Then our own GP had said: 'If there are any problems, you just call me', and then he gave us his private number – and then I called him and ... – well, he came very, very quickly." *(Respondent 5)

*"The home care services I could reach by phone and the nurse I could reach by phone, and our own GP I could reach by phone, I could call her at all hours." *(Respondent 7)

All the caregivers were involved in the physical treatment of the deceased. Only three took part in intimate hygiene, while the rest had help from the primary health care assistants.

*"The very first time she had to use a bedpan in bed, we talked about ... of course we could do it ourselves, but I only did it once then I called ...and they came very quickly."*(Respondent 7)

### Positive impact

The caregivers expressed a willingness to be active in the medical treatment and physical care and all of them would do it again.

*"Yes, I would, definitely I would, no doubt about that." *(Respondent 4)

All respondents had found themselves exceeding personal limits, but in doing so they found out that they were able to do more than they ever thought they could.

*"I crossed my own boundaries. I have always thought that dentures are very disgusting, but I could ... wearing gloves I could easily clean them. It was only the first time ... after that it was no problem." *(Respondent 1)

*"... but I readily admit ... if somebody had said to me the year before: "Now you will have to go through this and that", then I would probably have ... perhaps have said: "No, I can't do that", but you ****can***, *but there is a reaction afterwards." *(Respondent 2)

They were all satisfied that they could do more than they thought they could and that they had been able to do something for their dying loved one.

*"I am glad ... well, I am actually glad about ...not about the course, because that was terrible, but I am glad that it developed the way it did in the sense that I was able to help her." *(Respondent 4)

Their active participation in medical treatment and physical care gave them more freedom and the possibility to live a more natural family life.

*"Actually I was glad I could do it....then we had the day to ourselves, without all these comings and goings." *(Respondent 7)

They all expressed the importance of having been able to fulfil the wishes of their loved one

*"Well, I will put it like that: If I had not wanted to do it, and she had had to go somewhere else to die, then I think I would have been ashamed of myself for the rest of my life that I could not ... be the one who could be closest ... well, that, I think, I could not bear to live with." *(Respondent 4)

Furthermore, they all expressed a feeling of having been able to cope better in bereavement due to this involvement.

*"I think it makes it easier to get through the grief process, because – well – you have done all you could." *(Respondent 3)

*"Afterwards, I cannot blame myself for taking the easy way out and not being involved – so I am actually feeling quite pleased with myself." *(Respondent 7)

*"I also have to say, that if you don't just throw yourself into it – you really miss out on something..." *(Respondent 6)

*"It was really good, indeed, and you can do exactly as you want, and I hope ... yes I really hope that there are others who will take pleasure in it ... I hope that there are others who will do it." *(Respondent 6)

### Negative impact

Negative impacts on bereavement were found when the caregiver felt a lack of confidence and when they felt left alone. Furthermore, this had a negative impact on bereavement if there was bitterness toward the diagnosis or treatment period or if the caregiver felt powerless during the course of the disease. In this study only one respondent felt that they had been left alone during the palliative course at home. She explained this by the fact that she had felt left alone by the children of the deceased. She had expected one of them to take terminal leave to assist her in the last months. As no one did, she found herself alone too many hours with her husband.

*"On the other hand – it must have been so hard after all that I think – my children are never going to care for me – I want to go to hospice or nursing home. So, even though I am glad I did it, and satisfied with myself, it was too hard or else I wouldn't think like so, would I?" *(Respondent 1)

Feelings of bitterness and powerlessness were expressed by some, but all related to earlier stages in the disease history, e.g. during diagnosis or treatment in the hospital. Lack of confidence in the system related to the hospital setting, especially in relation to bad communication and inconsistency of professionals (especially changes of doctors, not being sure who was in charge of diagnosis and treatment) and time delays in diagnosis and treatment.

*"... but the first time ... you didn't see the same doctor twice, you know ... then it was a young lady who came who was very good and whom my wife liked, but then the weekend came and then she is gone." *(Respondent 5)

*"... ... I think it was very frustrating because when you needed to know something out there (at the hospital) ...well, you really had to insist, and once out there I was called absolutely hysterical, and then I looked at the doctor who came in and then I said: *'*let me tell you one thing, you walk out that door now and then you find the consultant NOW – and don't call me hysterical!' And then she kind of had to explain to me what she wanted." *(Respondent 6)

### Prerequisites

Three categories emerging from the analysis addressed prerequisites: Safety, Confidence and Dialog.

#### Safety

All the caregivers expressed that the feeling of safety for the patient and the caregiver was essential, emphasizing that professional back-up 24 hours a day was important.

*"It worked very well, and I really trusted those nurses I must say ...I really did... That's where I got my support from." *(Respondent 3)

*"Actually it was because the backup was good, that is ...well ... I didn't feel insecure at all." *(Respondent 7)

#### Confidence

It meant a lot to the caregivers that the professionals had confidence in the caregiver's abilities and this feeling of confidence enabled the caregiver to feel able to undertake the task.

*"His doctor never doubted my ability to undertake the task." *(Respondent 1)

#### Dialog

The caregivers felt it important to be asked if they could assist and what they would and could do. They also felt it important to have influence on decisions.

*"I think they (the professionals) should let the relative offer to help – ask them 'how do you feel about assisting with personal hygiene?' etc." *(Respondent 1)

"*I think they should ask, I think there are some people who won't ... will never get around to it, I think because I do think ...well, I think ... it would be so easy just to lean back and watch it all ... then you are not part of anything, you see." *(Respondent 4)

## Discussion

This study indicates that spouses' active involvement in palliative care has a positive effect on bereavement given that certain preconditions are fulfilled. The answers from the respondents were quite unanimous. It gives comfort in bereavement to have been active and able to help.

The most important preconditions for a positive effect are safety, e.g. professional back-up 24 hours a day, mutual confidence and a respectful dialog between caregiver and professionals about the tasks.

This project was prompted by questions from our daily clinical work asking to what extent to involve family caregivers who are able and willing to do extensive medical and physical care in palliative settings at home. It is often asked among professionals if it might be harmful to relatives coping with bereavement to take on the strain and burden of heavy practical involvement in palliative care. We feel that such discussions among our research team helped enhance our explorative awareness of the respondents' experiences and did not alter the results of the study.

AW chose two of her own patients' spouses, both having been active in medical and physical treatment. This might have led to altered results but as stated in the method section, these two interviews did not differ from the others in the analytic phase and were therefore included in the ultimate analysis. If a more profound analytic approach than Qualitative Description were chosen it might have had more importance. The use of GPs as mediators and henceforth their selection of respondents could also have altered the results and led to too early data saturation than a more open strategic sampling. On the other hand, we found that the information was so robust and uniform that we think our results are qualitatively representative of experiences among caregivers assisting with palliative care at home. Furthermore, this use of GPs as mediators helped us in the ethical considerations about contacting spouses one to three years after bereavement.

The study consist of a small number of respondents, but in an explorative and descriptive study like this we believe that the outcome is still applicable to daily clinical practice, but naturally estimates can not be given on the quantitative distribution of different experiences among spouses and further research will be required before we can give general guidelines to professionals in palliative home care.

Several studies have looked into the psychological implications on family caregivers in bereavement generally, without a focus on active involvement. It has been found that caregivers seem to cope better if sufficient support is provided during the palliative stage of disease at home. The most important issues seem to be good communication [5,6], well organized shared care [5], information about the disease [21] and nursing skills [6], accessibility to staff and their competence [8-12,22] and the caregivers own health and social network [6]. Home deaths are shown to be associated with both better bereavement response and better physical health post-bereavement than were inpatient deaths [10] and it has been shown to be important to have been doing something for the loved one who died [2-4]. This is in line with information from our caregivers. The manner in which the health care workers act towards the patient and relatives influences relatives' possibilities for involvement [21]. These implications were also found in our study.

Literature has shown that active involvement of family care givers could be harmful [2,13,23-25] and that the relatives experience an unspoken pressure from the professionals to be 'professionals' rather than loving relatives [5], but also that it makes bereavement easier [3,4,8,10,26]. In our study it seemed to be beneficial to be allowed to participate in medical treatment and physical care. It made life easier and less dependent on the schedule of professionals, which could otherwise have tied spouses to their home during specific times. Therefore active involvement may allow more room for being a loving relative, rather than limiting this room.

Hence, a natural question could be: "Why does active involvement work for some caregivers and not for others?" This question was not answered by earlier studies to our knowledge and it seems to be an under-researched topic. There is a lack of knowledge about how being active in treatment and care during the palliative course affects the quality of life and bereavement response of family caregivers compared to those not active in treatment and care. More research in this field is essential. However, we found some pre-conditions that seem important to enhance a positive outcome. Our study emphasizes the importance of communication, respect and accessibility.

It seems important that palliative health care workers and GPs receive good basic, vocational and continuing medical education in communicative skills in palliative care and in active dialog with caregivers.

## Conclusion

The results from this study identified important issues whenever spouses take an active part in medical treatment and physical care of critically ill patients in palliative care. The main categories were: Degree of involvement, Positive and Negative impact and Prerequisites. The prerequisites found for a positive outcome were Safety (24-hour back-up), Confidence (Professionals' confidence in the spouses' abilities) and Dialog (Spouses' influence on decision-making and being asked). The results question the previous research that active involvement of family care givers could be harmful and add preconditions to a positive outcome. More research into these preconditions is needed.

## Competing interests

The authors declare that they have no competing interests.

## Authors' contributions

AW participated in the design of the study, conducted the interviews and the analysis and drafted the manuscript.

FO and MAN participated in the design of the study, as supervisors in conducting the interviews and participated in discussions of categories.

All authors read, revised and approved the final manuscript.

## Pre-publication history

The pre-publication history for this paper can be accessed here:


